# Clinical differences between *Mycoplasma pneumoniae* pneumonia and *Streptococcus pneumoniae* pneumonia: a case control study

**DOI:** 10.3389/fped.2024.1409687

**Published:** 2024-07-19

**Authors:** Jinping Ruan, Zhou Fu, Linyan Ying

**Affiliations:** ^1^ Department of Pediatrics, Chongqing Red Cross Hospital (People’s Hospital of Jiangbei District), Chongqing, China; ^2^ Department of Respiratory Medicine, Children’s Hospital of Chongqing Medical University, National Clinical Research Center for Child Health and Disorders, Ministry of Education Key Laboratory of Child Development and Disorders, Chongqing Key Laboratory of Pediatrics, Chongqing, China; ^3^ Department of Pediatrics, Women and Children’s Hospital of Chongqing Medical University, Chongqing, China; ^4^ Department of Pediatrics, Chongqing Health Center for Women and Children, Chongqing, China

**Keywords:** *Mycoplasma pneumoniae* pneumonia, *Streptococcus pneumoniae* pneumonia, early diagnosis, independent predictive factor, clinical differences

## Abstract

**Introduction:**

*Mycoplasma pneumoniae* pneumonia (MPP) and *Streptococcus pneumoniae* pneumonia (SPP) are frequent causes of respiratory tract infection, the aims of the study were to explore the differences in clinical features between children with MPP and those with SPP.

**Methods:**

This retrospective study included admitted children who were diagnosed with MPP or SPP over 5 years from January 2015 to January 2020. Children with MPP were compared to children with SPP in terms of clinical features.

**Results:**

506 patients with MPP were compared to 311 patients with SPP in terms of clinical differences. The MPP group with a median age of 60 [29–89] months and the SPP group with a median age of 24 [10–40] months. Patients with MPP were older and had a higher occurrence of receiving antibiotics before admission, fever, dry cough, polypnea and diarrhea than patients with SPP (all *p* < 0.01). Patients with SPP were more likely to have wheezing, cyanosis and irritability (all *p* < 0.01). Laboratory findings in our study showed that there were significant differences between MPP and SPP patients in mean leucocyte count, neutrophil % (N%), lymphocyte % (L%), ALT levels, AST levels, LDH levels and incidence of accelerated procalcitonin (PCT) (all *p* < 0.01). Lower age, no dry cough, no polypnea, lower LDH levels, and higher PCT might lead to the diagnosis of SPP. Our study showed that age had a higher accuracy in predicting MPP than LDH levels, with an age >48.5 months shown to be an independent predictive factor for the early evaluation and identification of MPP.

**Discussion:**

In conclusion, patients with MPP and SPP usually present with fever, cough and some nonspecific symptoms. Our study showed that age, dry cough, polypnea, LDH levels, and PCT levels were independent predictive factors associated with MPP and SPP.

## Introduction

Community-acquired pneumonia (CAP) is a common infectious disease that is sometimes fatal and seriously threatens the health of children. CAP remains a significant cause of morbidity and death worldwide. *Mycoplasma pneumoniae* pneumonia (MPP) is one of the most frequent causes of respiratory tract infection, accounting for almost 30% of all cases of CAP (1, 2). Under normal circumstances, MPP is mainly a benign, self-limiting disease; however, the number of children with severe *Mycoplasma pneumoniae* pneumonia (SMPP) is increasing every year due to drug abuse, drug resistance, clinical misdiagnosis and missed diagnosis (3). Moreover, children with SMPP also have high rates of extrapulmonary organ dysfunction and macrolide resistance (4). In addition, *Streptococcus pneumoniae* (*S. pneumoniae*) is one of the most common leading pathogens of CAP, causing mild to life-threatening invasive diseases. Because of their few side effects and lower toxicity, β-lactam and macrolide antibiotics are used as first-line therapies in children (5). In recent years, the treatment of *Streptococcus pneumoniae* pneumonia (SPP) has become increasingly difficult due to the high levels of macrolide-resistant *S. pneumoniae*. Reports have shown that approximately 95% of *Streptococcus* strains show high resistance to erythromycin and clindamycin (6, 7). Therefore, it is difficult to manage SPP by medication with macrolide antibiotics as the first-choice drug. On the other hand, severe pneumonia associated with *S. pneumoniae* and *Mycoplasma pneumoniae* (*M. pneumoniae*) remains an important reason for ICU admission, and more than 50% of children with pneumonia in China die of *S. pneumoniae* (7). If acute MPP in children is not controlled in time, it may progress to bronchiolitis obliterans, which can greatly affect the quality of life of children (8). These facts indicate that the identification of MPP and SPP in the early stage could be important. To our knowledge, there are already some scoring systems that propose a differential diagnosis between MPP and some bacterial pneumonias for selection of the appropriate antibiotics for the treatment of CAP (9). In fact, there are many situations in which we cannot accurately distinguish between the two groups of patients. When MPP presents as lobar pneumonia, the patient has similar clinical manifestations and imaging features to SPP, even those experienced radiologists and pediatricians are difficult to distinguish between the two diseases.

Thus, the purpose of the present study was to determine whether we could differentiate between pneumonia caused by *M. pneumoniae* and *S. pneumoniae* on the basis of epidemiological and clinical manifestations and laboratory results before infection was serologically confirmed. The findings from our study are anticipated to provide evidence-based medical guidelines for early diagnosis, enabling the treatment of CAP caused by *M. pneumoniae* and *S. pneumoniae*, thereby leading to improved prognosis.

## Methods

### Study population

The study was retrospective and included all children with clinical evidence of CAP caused by *S. pneumoniae* or *M. pneumoniae*. The patients were hospitalized at the Children’s Hospital of Chongqing Medical University over 5 years (from January 2015 to January 2020). The inclusion criteria were pneumonia defined as a new pulmonary infiltrate on the chest radiograph or chest CT and either a positive specific polymerase chain reaction (PCR) test for *M. pneumoniae* on respiratory specimens (noninvasive samples or bronchoalveolar lavage) or identification of *S. pneumoniae* in respiratory specimens. The diagnoses of *M. pneumoniae* and *S. pneumoniae* were all in line with the diagnostic criteria for *M. pneumoniae* and *S. pneumoniae* of the Respiratory Group of the Pediatric Branch of the Chinese Medical Association (10). Patients were excluded if they had any of the following: hospital-acquired pneumonia; infection by other respiratory pathogens; infection of other systems; diagnosed connective tissue disease or immune deficiency; prior organ or bone marrow transplantation; congenital heart disease, bronchiectasis and other basic diseases; or incomplete main outcomes.

### Data collection

We used a standardized form for data collection and extracted the following data through the medical records system: age, sex, onset season, duration of hospitalization, clinical manifestations, and laboratory results.

### Statistical analysis

The measurement data showed a skewed distribution, which was represented by median (quartile distance) M (P25–P75), and the comparison between groups was performed by Mann‒Whitney *U* test. The enumeration data were represented by the number of cases (percentage) *n* (%), and the comparison between groups was performed by the *χ*2 test or Fisher’s exact probability method. Logistic regression was used to identify independent predictive factors associated with *M. pneumoniae* and *S. pneumoniae*, and variables with *p* < 0.1 in the univariate logistic analysis were included in the multivariate logistic regression analysis. Stepwise forward selection (likelihood ratio) was used in the multivariate analysis to create a logistic proportional hazards model to identify the independent predictive factors of MPP and SPP. The inclusion criterion for factors was *p* < 0.05. All statistical analyses were performed with the R.4.2.0 for Windows software package.

## Results

### Demographic characteristics

**Figure 1:**
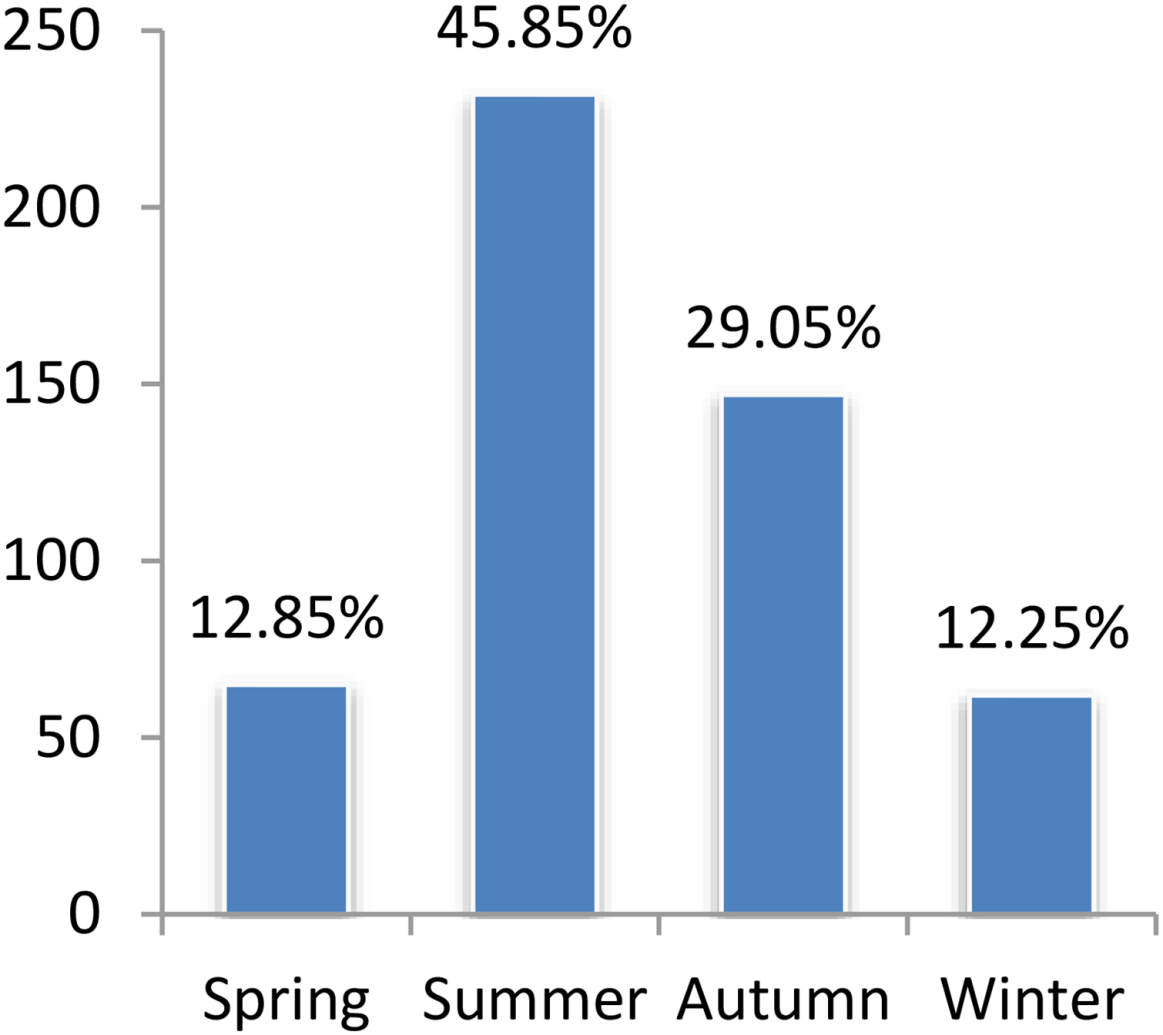
Distribution MPP cases by season.

**Figure 2:**
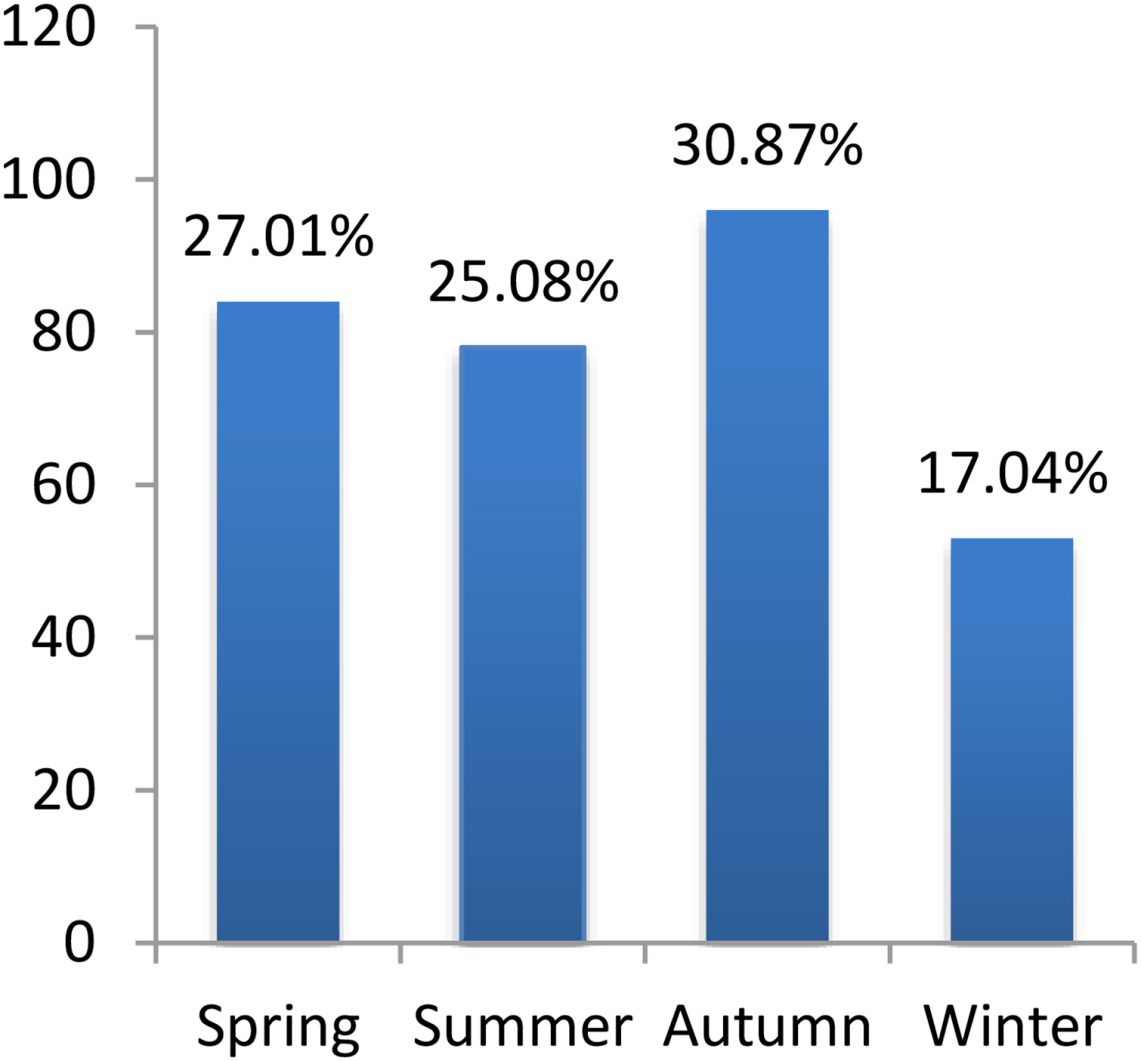
Distribution SPP cases by season.

We included 506 patients with MPP; 283 were male, and 223 were female, with a median age of 60 [29–89] months. We also included 311 patients with SPP; 180 were male, and 131 were female, with a median age of 24 [10–40] months. As depicted in Figures 1, 2, MPP and SPP could occur throughout the year, with obvious seasonality. MPP was common in summer and autumn, and SPP was common in spring and autumn. Significant differences were found in morbidity in different seasons (*p* < 0.001, *p* = 0.005, respectively). Details of patients with MPP and SPP are presented in Table 1. The majority of patients were males in both study groups (*p* = 0.636). The mean age in the MPP group was significantly higher than that in the SPP group (*p* < 0.001).

**Table 1:** Basic details of patients with MPP and SPP.

Charactristics	*M. pneumoniae*	*S. pneumoniae*	*P* value
	(*N* = 506)(%)	(*N* = 311)(%)	
Severe	77 (15.2%)	40 (12.9%)	
Sex			0.636
Males	283 (55.9%)	180 (57.9%)	
Females	223 (44.1%)	131 (42.1%)	
Age(m)	60 (29.25,89)	24 (10,40)	<0.001
Average hospitalization(d)	7 (6,8)	7 (6,8)	0.315
Received antibiotics before admission	472 (93.9%)	269 (86.5%)	0.002

### Clinical features

There was a higher incidence of receiving antibiotics before admission to hospital in MPP patients than in SPP patients (472/506 vs. 269/311, *p* = 0.002). However, there was no significant difference in the proportion of severe pneumonia and the average duration of hospitalization in the two groups (*p* > 0.05). Patients in both groups had similar signs and symptoms upon admission to the hospital (Table 2). The most common symptoms in patients with MPP and SPP infection were fever (88.70% vs. 97.96%), gastrointestinal tract symptoms (70.20% vs. 72.99%), poor appetite (57.90% vs. 59.81%), and high temperature (T ≥ 39.5°C) (42.10% vs. 35.05%), respectively. Patients with MPP were more likely to have a fever, a dry cough and shortness of breath (*p* < 0.05) but less likely to experience wheezing, cyanosis and dysphoria upon admission (*p* < 0.05). In terms of gastrointestinal tract symptoms, diarrhea (*p* < 0.05) was more commonly associated with MPP. Table 3 reports the pulmonary signs of these patients in both groups. In patients with MPP, crackles were more likely to be heard (237/311 vs. 309/506, *p* < 0.001) and rhonchi were less likely to be heard (136/311 vs. 97/506, *p* < 0.001). All signs in the patients with SPP and MPP were more likely to be bilateral (230/311 vs. 226/506, *p* < 0.001) and less likely to be unilateral (18/311 vs. 92/506, *p* < 0.001).

**Table 2:** Symptoms and signs in patients with MPP and SPP.

Symptoms and signs	*M. pneumonia* (*N* = 506)		*S. pneumoniae* (*N* = 311)		*P* value
	No.	%	No.	%	
Fever	449	88.74	235	75.56	<0.001
Temperature （T ≥ 39.5°C)	213	42.10	109	35.05	0.054
Dry cough	84	16.60	20	6.43	<0.001
Wheezing	100	19.80	148	47.59	<0.001
Polypnea	152	30.00	72	23.15	0.039
Cyanosis	142	28.10	126	40.51	<0.001
Neurological symptoms	184	36.40	127	40.84	0.229
Bad mental state	172	24.00	101	32.48	0.712
Irritable	16	3.20	40	12.86	<0.001
Convulsions	3	0.60	6	1.93	0.091
Gastrointestinal tract symptoms	355	70.20	227	72.99	0.430
Poor appetite	293	57.90	186	59.80	0.644
Vomiting after coughing	125	24.70	85	27.30	0.452
Diarrhea	71	14.00	23	7.40	0.006

**Table 3:** Symptoms and signs in patients with MPP and SPP.

Signs	*M. pneumoniae* (*N* = 506)		*S. pneumoniae* (*N* = 311)		*P* value
	No.	%	No.	%	
Crackles	309	61.07	<0.001	76.21	<0.001
Rhonchi	97	19.17	<0.001	43.73	<0.001
Signs			<0.001		<0.001
Bilateral	226	44.66	<0.001	73.95	<0.001
Unilateral	92	18.18	<0.001	5.79	<0.001
None	188	37.15	<0.001	20.26	<0.001

### Laboratory results

Table 4 shows the laboratory results of the 2 groups. There were significant differences in the mean leucocyte count, N%, L%, ALT levels, AST levels and LDH levels between patients with MPP and those with SPP (*p* < 0.05). Patients with SPP had higher levels of mean leucocyte counts [10.94(7.82,14.79)*10^9^/L], L% [42(26,55)%], ALT levels [16(12.03,22.88) U/L] and AST levels [34.9(27.35,43.45) U/L] than those with MPP (7.745(5.94,10.09)*10^9^/L, 32(24,41)%, 16(12.03,22.88) U/L, and 32(27.1,39) U/L, respectively). Patients with MPP had higher levels of N% [62(52.25,70)%] and LDH levels [305.5(260,363) U/L] than those with SPP (52(39,69)% and 295.4(245.5,346) U/L, respectively). A higher proportion of SPP patients was recorded to have accelerated PCT than MPP patients (80/311 vs. 43/506, *p* = 0.001). A total of 172 patients (55.3%) from the SPP group and 263 (52.0%) from the MPP group had normal CRP levels (*p* = 0.393).

**Table 4:** Laboratory findings in patients with MPP and SPP.

Laboratory findings	*M. pneumoniae* (*N* = 506)	*S. pneumoniae* (*N* = 311)	*P* value
Leucocytes(^a^10^9^/L)	7.745 (5.942,10.092)	10.94 (7.815,14.790)	<0.001
N%	62 (52.25,70)	52 (39,69)	<0.001
L%	32 (24,41)	42 (26,55)	<0.001
ALT (U/L)	16 (12.03,22.88)	17.60 (13.30,25.40)	0.004
AST (U/L)	32 (27.1,39)	34.9 (27.35,43.45)	0.016
LDH (U/L)	305.5 (260,363)	295.4 (245.5,346)	0.009
CRP↑^a^	243 (48.0%)	139 (44.7%)	0.393
PCT↑^b^	43 (8.5%)	80 (25.7%)	0.001

^a^ CRP↑: CRP > 8 mg/L.

^b^ PCT↑: PCT > 0.05 ng/ml.

### Predictive factors

The potential predictive factors for SPP identified in univariate and multivariate analyses with statistical significance (*p* < 0.05) are shown in Table 5. The final model of the multivariate logistic regression studies showed that age, dry cough, polypnea, LDH levels and levels of PCT were independent predictive factors associated with MPP and SPP. Receiver operating characteristic (ROC) curve analysis was used to determine the accuracy of age and serum LDH levels as predictors of SPP (Table 6). We considered LDH to have a lower prediction value due to its low sensitivity and specificity. The cutoff level, sensitivity, specificity and area under the ROC curve (AUC) for age were 48.5 months, 88.1%, 60.1%, and 0.785, respectively.

**Table 5:** Potential predictive factors from univariate and multivariate analyses.

Variables	Univariate analysis		Multivariate analysis	
	*P* value	OR(95% CI)	*P* value	OR(95% CI)
Age	<0.001	0.96 (0.95–0.97)	<0.001	0.96 (0.95–0.97)
Dry cough	0.001	0.35 (0.20–0.56)	0.043	0.52 (0.27–0.96)
Polypnea	0.033	0.70 (0.51–0.97)	0.004	0.51 (0.32–0.80)
LDH (U/L)	0.013	0.998 (0.997–0.999)	<0.001	0.996 (0.994–0.998)
PCT↑	<0.001	3.73 (2.51–5.62)	<0.001	6.08 (3.51–10.80)
Received antibiotics before admission	0.001	0.46 (0.29–0.74)		
Fever	0.001	0.39 (0.27–0.57)		
Wheezing	0.001	3.69 (2.70–5.05)		
Cyanosis	<0.001	1.75 (1.30–2.35)		
Irritable	<0.001	4.52 (2.53–8.45)		
Diarrhea	0.005	0.49 (0.29–0.79)		
Crackles	0.001	2.04 (1.49–2.81)		
Rhonchi	0.001	3.28 (2.40–4.50)		
Signs-bilateral	<0.001	3.04 (2.17–4.29)		
Leucocytes (*10^9^/L)	<0.001	1.16 (1.12–1.21)		
N%	<0.001	0.97 (0.96–0.98)		
L%	<0.001	1.03(1.02–1.04)		

**Table 6:** Predictive value of age and LDH levels in patients with SPP.

Independent factors	Cutoff Value	Sensitivity	Specificity	AUC	*P* value	95% CI	
						Lower	Upper
Age(m)	48.5	0.881	0.601	0.785	<0.001	0.754	0.815
LDH (U/L)	336.5	0.717	0.377	0.555	0.009	0.514	0.595

## Discussion

In total, 506 patients with MPP and 311 patients with SPP who had been hospitalized at the Children’s Hospital of Chongqing Medical University were included. Previous studies have shown that *M. pneumoniae* and *S. pneumoniae* are the major causative agents of CAP in children (11). According to the WHO, pneumonia accounts for 16% of all deaths in children under 5 years old, with *S. pneumoniae* being the most common cause of bacterial pneumonia (5).

In our study, a higher incidence of MPP was recorded in summer and autumn, which was the same as a previous study in Nanjing, China (12). However, *S. pneumoniae* CAP was recorded as common in spring and autumn, which differed from some reports. Previous studies have shown that *M. pneumoniae* infections are uncommon in children under the age of 5, with the maximum frequency occurring between the ages of 5 and 15 (2, 13), and other studies have shown that children aged 2–5 years are more susceptible to *M. pneumoniae* infections (14). Consistent with some other studies (11), our results show that the highest incidence of MPP was among preschool children aged around 5 years old, while the highest incidence of SPP was in infants and young children aged around 2 years old.

In both groups of patients, males were affected more often than females, which correspond to the findings of some previous studies (6, 13). We observed that 93.9% of MPP and 86.5% of SPP patients received antibiotics before admission, while there was no significant difference in the proportion of severe pneumonia and the average duration of hospitalization in both groups.

We observed several differences between the symptoms and signs produced by MPP and SPP. It has been reported that MPP usually presents with fever, cough, diarrhea, and some other nonspecific symptoms and that the clinical symptoms, laboratory data and radiologic findings of MPP are different from those of other bacterial pneumonias (15–17). Previous studies have demonstrated that both *M. pneumoniae* and *S. pneumoniae* are pathogens that cause persistent wheezing, which causes an inflammatory stimulus in children with persistent wheezing (18, 19). Acute *M. pneumoniae* infection can cause not only acute attacks in children with asthma but also wheezing in nonasthmatic children (20). In our study, the patients with MPP were more likely to have a fever, dry cough and diarrhea than patients with SPP but less likely to have wheezing, cyanosis and irritability.

Both groups of patients were observed as being more likely to have crackles and less likely to have rhonchi in the lungs. A higher proportion of SPP patients had signs, either crackles or rhonchi. In addition, 73.95% of SPP and 44.66% of MPP patients had bilateral signs, while 20.26% of SPP and 37.15% of MPP patients had no signs in the lungs. In our study, 15.2% of MPP patients and 12.9% of SPP patients were diagnosed with severe pneumonia. These data may suggest that some children with SMPP had few signs in the lungs, which led to misdiagnosis and missed diagnosis.

In recent years, hematological markers have gradually begun to be used in the clinical diagnosis and prognosis of infectious diseases, and the neutrophil to lymphocyte ratio (NLR) may reflect the degree of inflammation and the state of immunity (21). A previous study reported that the NLR may have clinical value in the differential diagnosis of bacterial pneumonia, and the NLR value was significantly higher in children with bacterial pneumonia than in children with MPP (22). Analysis of the laboratory findings in our study showed that there were significant differences between the two groups in mean leucocyte count, N%, L%, ALT levels, AST levels, LDH levels and incidence of accelerated PCT. Similar to a previous study, the perpheral leucocyte count of the patients with MPP is usually normal at less than 10,000/UL (17). The higher leucocyte counts and PCT levels may suggest that the inflammation of *Streptococcus pneumoniae* pneumonia is more serious than that of *Mycoplasma pneumoniae* pneumonia. Although significant differences in inflammatory markers were observed between the two groups, these markers were not found to be adequate for predicting SPP. This may be due to the large overlap of serum inflammatory markers between groups or the inherent characteristics that these markers are nonspecific and reflect the level of whole-body inflammation (23).

In our study, age, dry cough, polypnea, LDH level and PCT level were independent predictive factors associated with MPP and SPP. Younger age, no dry cough, no polypnea, lower levels of LDH, and higher PCT might lead to a diagnosis of SPP. MPP can lead to liver damage in patients, which may be directly caused by *M. pneumoniae* or may result from the liver’s immune response to *M. pneumoniae*. LDH can be released into the blood when hepatolysis or cell membrane damage of hepatocytes, resulting in significantly elevated LDH levels in children with SMPP. Unlike previous studies have shown that serum LDH levels may indicate the severity and prognosis of pneumonia (24–26), our results showed that LDH was associated with *M. pneumoniae* infection. However, the area under the ROC curve of serum LDH levels in our study was smaller, and the prediction value was smaller. Previous studies suggest that age may be a main factor associated with *M. pneumoniae* infection, but the underlying mechanisms remain unclear (6, 27). In our study, age > 48.5 months was an independent predictive factor for early evaluation and identification of MPP. This study provides a possible theoretical basis for distinguishing between MPP and SPP.

## Conclusions

In summary, there are many differences between *Mycoplasma pneumoniae* pneumonia and *Streptococcus pneumoniae* pneumonia. Age, dry cough, polypnea, LDH level and PCT level were independent predictive factors associated with MPP and SPP. An age > 48.5 months could be a possible predictor for the early evaluation and identification of MPP. This is the first study to elucidate the differences between MPP and SPP, providing a theoretical basis for early identification. There are several strengths of our study. First, to the best of our knowledge, this is the first study to analyze the differences in clinical features between children with MPP and those with SPP. Second, our study identified predictive factors to distinguish between children with MPP and those with SPP. However, the study still has some limitations. First, it is a retrospective study, which appears to have a selection bias. Second, the presence of other pathogens could not be detected due to shortcomings in the existing detection technology. Third, the distribution of the number of patients among the two groups could not be balanced, which could have influenced the statistical analysis. Fourth, the cases in our study were all children from southwestern China, and multicenter study with a large sample size is required in future studies.

## Data Availability

Based on the original data of this study involving patient privacy and ethical issues, we can provide data upon reasonable request. Requests to access datasets should be sent directly to Ying Linyan, 481057@cqmu.edu.cn.
